# Management of Fused Primary Anterior Teeth: A Case Series

**DOI:** 10.1155/crid/6187804

**Published:** 2025-05-14

**Authors:** Mohamed Salah Shalaby, Osama Ibrahim El Shahawy

**Affiliations:** ^1^Dental Public Health, Ortho and Pediatric Dentistry Department, Faculty of Dentistry, Future University in Cairo, Cairo, Cairo Government, Egypt; ^2^Pediatric Dentistry Department, Faculty of Dentistry, Cairo University, Cairo, Cairo Governorate, Egypt

## Abstract

Fusion is a developmental dental anomaly that may affect both dentitions. The condition occurs during the developmental stage when two different tooth buds fuse. Depending on the degree of fusion, teeth may be diagnosed with fusion, gemination, or macrodontia. This defect is typically described as primary double teeth. The main clinical problems associated with primary double teeth include dental caries, crowding, occlusal discrepancies, and poor aesthetics. This report presents the clinical experience of managing 13 primary fused anterior teeth. The aim was to preserve and restore the decayed teeth while maintaining function and aesthetics. Treatment varied from the application of preventive measures to the separation of fused teeth. Clinical and radiographic diagnoses were used to determine the appropriate treatment plan. Decay removal was followed by restoration using either composite or zirconia crowns. Some teeth required pulp treatment before final restoration; therefore, pulpectomy was performed, and the canals were sealed with a calcium hydroxide and iodoform mix. Evaluation criteria included clinical success, gingival health, function, and aesthetic preservation. Follow-up periods ranged from 1 to 4 years. The follow-up of the presented cases demonstrated that the selected preventive, restorative, and surgical approaches successfully retained the affected teeth in a stable and healthy condition.

## 1. Introduction

Fusion of teeth is a developmental anomaly that may affect both primary and permanent teeth. The etiology of this condition is unclear, but it may be related to physical pressure on the developing follicles [1, 2]. The condition arises during the developmental stage when two different tooth buds fuse into one. Complete fusion into a single root canal system is likely if fusion occurs before the calcification phase. However, root fusion with separate canals is typically the result if it occurs later.

This developmental defect is usually described as primary double teeth (PDT) or primary fused teeth (PFT) [3, 4]. According to the morphological classification of Ben Salem et al., fusion can be categorized into four types: Type I, bifid crown with a single root; Type II, large crown with a large root; Type III, two fused crowns with double conical roots; and Type IV, two fused crowns with two fused roots [5]. The prevalence of fusion in the primary dentition has been reported to be 0.5%–1%, which is higher than in the permanent dentition (0.01%–0.2%) [6], with a greater tendency to occur in the mandibular arch [4]. It can be bilateral or unilateral [7], with no significant difference reported between males and females [8].

The primary clinical problems associated with fused teeth include dental caries, crowding, occlusal discrepancies, and poor aesthetics. The need for intervention is determined by the patient's needs and a comprehensive assessment of the affected and surrounding teeth. Factors such as morphology, position, degree of fusion, and root canal configuration should be considered when designing a treatment plan for these cases [9]. Possible treatment options include no intervention, preventive measures, orthodontic treatment, restorative treatment, and surgical modification with or without hemisection or extraction.

## 2. Aim of the Case Series Report

This report presents the clinical experience of managing 13 different fusion cases. Treatment was supported by a follow-up period ranging from 1 to 4 years.

This case series was approved by a retrospective study statement from the research ethics committee of the Faculty of Dentistry, Cairo University, Egypt, on June 23, 2023.

**Figure 1:**
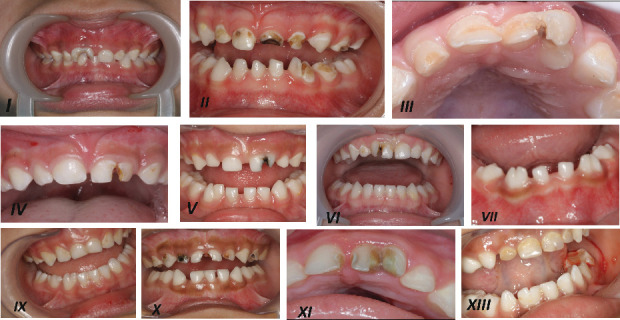
Clinical picture of most of the described patients in Table 1.

Thirteen patients were included in this case series, presenting with 15 fused teeth (five maxillary, six mandibular, and two in both arches) (Figure 1 and Table 1). Table 1 summarizes all examined conditions, types of intervention, follow-up periods, and results.

In all treated cases, the following materials were used:

• Local anesthesia: lidocaine HCl 2% with epinephrine 1:100000 (Septodont, Canada).
• Primary teeth root canal-filling material: Metapex Plus (Meta Biomed, Korea).
• Packable self-cured glass ionomer restorative: GC Fuji IX capsules (GC Dental, Japan).
• Pediatric prefabricated zirconia crown: NuSmile (Houston, TX, United States).
• Fluoride varnish (applied as a preventive measure in cases with no treatment): Enamel Pro Varnish (Premier, PA, United States).

### 2.1. Case 1

She was a 4-year, 10-month-old female presented with a complaint of a disfigured upper incisor. She was medically free, with no family history of dental anomalies. Clinical examination revealed coronal fusion between the maxillary right primary central incisor and a supernumerary tooth. A C-shaped cavity was evident in the fusion area; according to the morphological classification, it was diagnosed as Type III.

**Figure 2:**
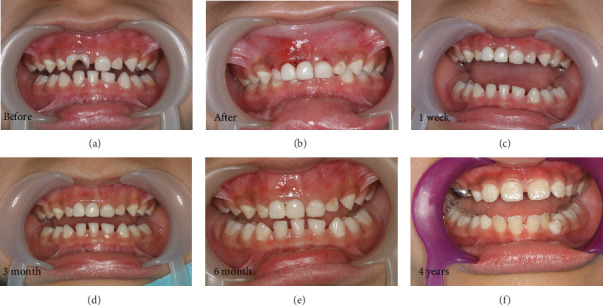
(a–f) Sequence of treatment of maxillary fusion by NuSmile zirconia crowns from preoperative to complete eruption of permanent incisors, 4 years follow-up.

**Figure 3:**
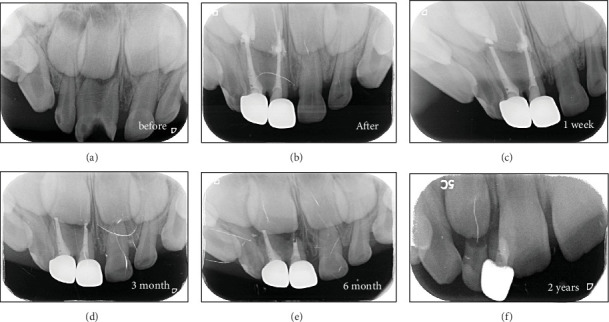
Sequence of treatment with follow-up till permanent teeth eruption. (a) Coronal fusion. (b–e) Gradual root resorption and Metapex melting. (f) Permanent tooth eruption.

Her lower left primary central and lateral incisors were fully fused (Figure 2a). Radiographic examination of the upper fusion revealed coronal fusion with two separate roots and distinct root canals (Figure 3a). The gingival line appeared higher than that of the adjacent teeth, with a loss of the interdental papilla. After discussing the treatment options and obtaining verbal consent from the parents, local anesthesia was administered. Following rubber dam application, a full pulpectomy was performed on teeth (#51 and #51⁣′), and the canals were filled with root canal filling material. The coronal portions of both teeth were then restored with a packable self-cured glass ionomer restorative. Each tooth was finally restored with a pediatric prefabricated zirconia crown (Figures 2b and 3c). The selected crown sizes were chosen to accommodate the available space and match the contralateral tooth.

The patient was followed up at 1 week, 1 month, 3 months, 6 months, 2 years (shedding time), and 4 years (full eruption and alignment of the permanent anterior teeth). At the 1-week follow-up, the gingiva appeared healthy, with the interdental papilla re-established and a slight difference in gingival height compared to the contralateral tooth. Both treated teeth exhibited clinical success throughout the follow-up period, with fully established function and aesthetics and no signs of infection or crown loss (Figures 2b, 2c, 2d, 2e, and 2f).

Radiographically, the immediate postoperative x-ray showed complete radicular filling of both roots. Minimal apical resorption was observed at the 3-month follow-up. At 6 months, resorption was evident in one-third of the root. By the 2-year follow-up, one tooth had shed naturally, while the other required removal to allow the normal eruption of the permanent central incisor (Figures 3b, 3c, 3d, 3e, and 3f).

After 4 years of the initial intervention, the permanent teeth were fully erupted with normal gingival contour and alignment (Table 1—I Kw).

### 2.2. Case 2

**Figure 4:**
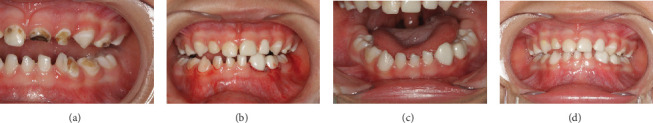
(a) Mandibular fusion between deciduous 72/73. (b–d) Case management with zirconia crown follow-up.

**Figure 5:**
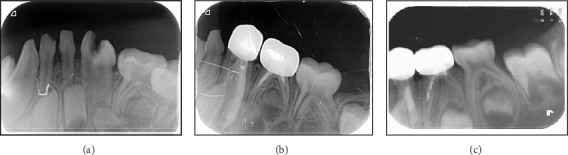
Treatment sequence follow-up. (a) Fusion 72/73 with two pulp canals. (b, c) Pulpectomy with Metapex filling materials and zirconia crown 1 month and 2 years, respectively.

She was a 3-year-old female presented with a complaint of early childhood caries. Examination revealed generalized decay in her upper and lower primary teeth. The mandibular left lateral incisor (#72) was fused with the neighboring canine (#73), forming a large, disfigured, and decayed tooth (Figure 4a). Radiographic examination revealed complete coronal and radicular fusion (Figure 5a), and according to the morphological classification, it was diagnosed as Type IV.

After discussing the case with the parents and obtaining their consent, local anesthesia was administered, and a rubber dam was applied. Caries were removed, a full pulpectomy was performed, and the canal was filled with a root canal-filling material. The coronal portion was then restored with packable glass ionomer and prepared to receive a relatively large zirconia crown (an upper crown was used) to accommodate the width of the enlarged tooth. An occlusion check was performed to exclude any occlusal interference with the opposing dentition (Figure 4b). The remaining decayed teeth were treated in subsequent sessions.

Follow-up clinical examination revealed excellent gingival integration, with full retention of the crown throughout the 4-year follow-up period (Figure 4). Radiographically, the root was fully filled, with some evident voids in the material, and normal development of the successor and neighboring teeth was observed (Figures 5) (Table 1—II Kw).

### 2.3. Case 3

**Figure 6:**
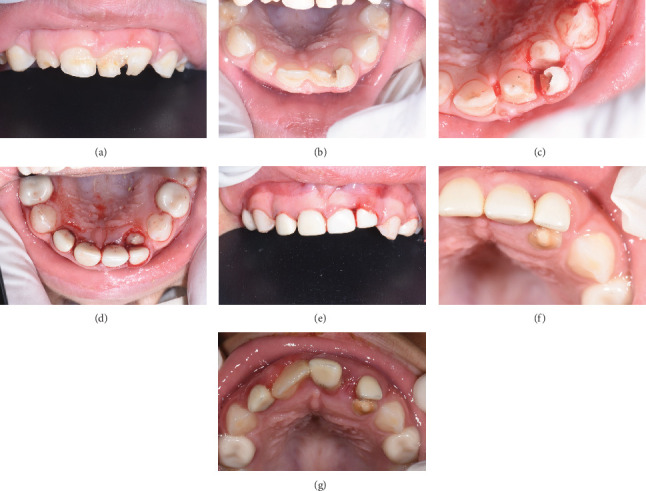
Case 3 triple fusion (Type Ia). (a, b) Preoperative picture. (c) After pulpectomy and composite restoration. (d, e) Immediate postoperative after zirconia crown insertion. (f) 1 year follow-up. (g) 3.5 years follow-up.

**Figure 7:**
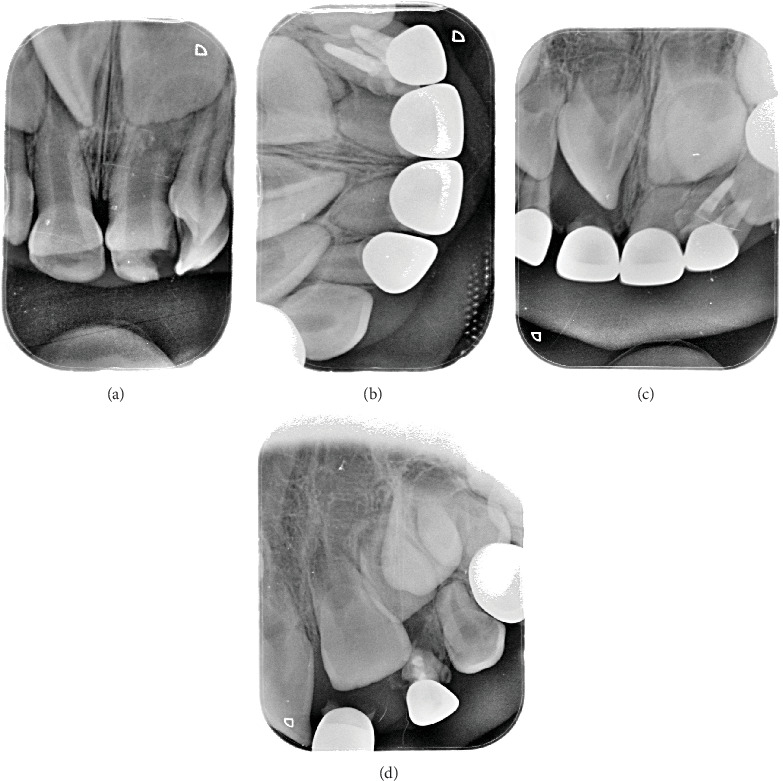
Triple fusion Type Ia. (a) Preoperative x-ray. (b) Postoperative x-ray. (c) 2.5 years follow-up. (d) 3.5 years follow-up.

He was a 3-year-old medically free male presented with multiple decayed primary teeth. Examination revealed a fusion of the crowns of the upper left central and lateral incisors and a third supernumerary tooth positioned palatally to both (Figure 6a,b). The condition was classified as Type Ia fusion, a fusion of two normal teeth with a supernumerary tooth, exhibiting three pulp chambers and three root canals due to fusion [10]. The three fused teeth were affected by decay, with a buccal bulge resulting from malalignment. A periapical radiograph confirmed the presence of three roots with three root canals (Figure 7a).

The proposed treatment plan involved separating the crowns, extracting the palatal tooth, and restoring #61 and #62 with crowns after caries removal. Due to a lack of patient cooperation, treatment under general anesthesia was recommended. After obtaining parental consent, all decayed teeth were treated. Upon caries removal, #62 and #62⁣′ were found to be pulpally involved. An attempt was made to separate the triplet; however, intraoperatively, it was determined that root fusion was present, contrary to the appearance in the two-dimensional radiographic image.

The intraoperative decision was to either extract or retain the fused triplet with modifications. After consulting the parents, it was decided that the fused teeth should be preserved. Pulpectomy was performed on #62 and #62⁣′, filling the canals with root canal filling material. The crowns were restored with packable glass ionomer, followed by the placement of zirconia crowns on #51, #52, #61, and #62, while #62⁣′ was restored with packable glass ionomer as the final restoration (Figures 6, 6, 6, and 6). The child was followed up for 3 years and 6months (Figures 6 and 7), during which the triplet area remained clinically stable, with healthy gingival tissues till time of shedding (Figure 6).

Radiographically, the immediate postoperative image showed good root canal filling with normal resorption and development. However, the 3-year follow-up radiograph revealed an evident supernumerary tooth related to #21, necessitating another round of intervention (Figure 7c) (Table 1—III Egy).

## 3. Discussion

Fusion is a condition in which teeth unite during the morphological stage. The degree of union determines whether the teeth are diagnosed as fused, geminated, or macrodontia. Teeth are classified as fused if the junction is limited to the crown, with two distinct roots. In gemination, the junction is nearly complete, with one root and a bifid crown, while in macrodontia, the teeth are fully united, forming a single large tooth [11].

The junctional line typically contains a deep, narrow groove, an inaccessible and retentive area that can lead to plaque accumulation and dental decay.

In this case series, the prevalence of mandibular fusion was found to be higher than maxillary fusion, consistent with the findings of Açıkel and İbiş [4]. The large tooth size, abnormal shape, bifid crown, and tooth malalignment can affect a child's appearance, potentially disrupting psychological well-being and leading to introversion. Uneven root resorption is frequently associated with fusion, which may interfere with the normal shedding process [2]. Proper management can enhance a child's aesthetic, functional, and psychological status while preventing anticipated malocclusions [12].

Factors such as crown position, inclination, occlusal interference, and root canal complexity dictate the most suitable restoration type when designing a treatment plan. Each dental anomaly case has unique characteristics that may not be comparable to others, as Costa et al. noted [11].

A review of the available literature showed that many authors have described cases of different types of fusion with only radiographic follow-up [13–21]. Others reported topical fluoride application as an intervention [22–24]. Furthermore, fissure sealant application for the grooves at the fusion line, followed by fluoride treatment, has been reported [25–28]. More advanced interventions range from groove filling with composite [23, 26, 29] to pulpectomy followed by composite restoration in pulp-involved teeth [7, 30–32], ultimately leading to extraction in some cases [14, 31].

The interventions presented in this case series were based on clinical and radiographic evaluations, considering patient needs. Treatment ranged from preventive measures to restorative solutions to address irregular crown shape and decay around developmental defect areas. The composite was used in one of the presented cases as a restorative alternative to reconstruct the normal anatomical crown form and restore decay. However, this restoration did not retain its integrity over the follow-up period, as chipping and discoloration were evident.

Zirconia crowns were alternatively used to provide a reliable and durable solution for restoring decay, function, and aesthetics while ensuring superior gingival integration. Pulp exposures resulted from crown separation in some cases, while in others, they were due to deep carious lesions. All pulp-involved teeth were treated with pulpectomies followed by crown placement. This approach ensures a long-term solution to mitigate the sequelae of developmental anomalies and decay. In some cases, the gingival line of crowned teeth appeared slightly different from that of the unaffected neighboring teeth, likely due to differences in size, position, and inclination. However, this variation was considered within the normal aesthetic range.

## 4. Conclusion

PFT or PDT is frequently observed in primary dentition, with a higher tendency to occur in the mandibular arch. Caries formation is commonly encountered at the junction between fused teeth. Through regular checkups, parents should be aware of the condition to prevent its potential complications. Pediatric zirconia crowns offer an effective aesthetic option for restoring these dental anomalies.

Why this paper is important to pediatric dentists:

• presents different management approaches for pediatric fused primary anterior teeth using zirconia crowns
• enhances awareness among pediatric dentists and general practitioners regarding the management of fused primary teeth.
• improves the aesthetic appearance of children with fused primary anterior teeth

## Figures and Tables

**Table 1:** Summary of 15 fused teeth conditions in 13 cases (five maxillary, six mandibular, and two in both arches). Eleven cases were treated in private practice in Kuwait (Kw) and two were treated in private practice in Egypt (Egy).

**#**	**Arch**	**Teeth**	**Diagnosis**	**Type of intervention**	**Follow-up**	**Clinical**	**X-ray**
I Kw	Max and Mand	51/51⁣′ and 71/72	Crown and root fusion with two root canals (max) and macrodontia (Mand)	Zirconia crowns	2 years	Success	Success
II Kw	Mand	72/73	Crown fusion with one root with two root canals	Zirconia crowns	4 years	Success	Success
III Egy	Max	61/62/61⁣′	Triple crown fusion with three separate roots	Zirconia crowns and composite	3.5 years	Success	Success
IV Kw	Max	61/62	Crown fusion with two separate roots	Zirconia crowns	2 months	Success	Slight root resorption in 61
V Kw	Max and Mand	61/61⁣′ and 82/83	Crown fusion with two separate roots	Zirconia crowns (maxillary) and preventive (mandibular)	18 months	Success	Success
VI Kw	Max	51/51'	Crown and root fusion with two root canals	Zirconia crowns	9 months	Success	Success
VII Kw	Mand	72/73 and 82/83	Bilateral gemination	Prevention	n/a	n/a	n/a
VIII Kw	Mand	72/73	Gemination	Prevention	n/a	n/a	n/a
IX Kw	Mand	82/83	Gemination	Prevention	n/a	n/a	n/a
X Kw	Mand	81/82	Macrodontia	Prevention	n/a	n/a	n/a
XI Egy	Max.	61/62	Crown fusion with two roots	Composite	6 months	Success	Success
XII Kw	Max.	51/52	Crown fusion with two roots	Prevention	6 months	Success	Success
XIII Kw	Mand	81/82	Crown fusion with two roots	Prevention	3 months	Success	Success

## Data Availability

The data that support the findings of this study are available on request from the corresponding author. The data are not publicly available due to privacy or ethical restrictions.
